# Bilateral Anteverted Conchal Bowls: Surgical Correction of a Rare Anomaly

**DOI:** 10.7759/cureus.19841

**Published:** 2021-11-23

**Authors:** Freya Scutt, Ahmed Mahmood, Jennifer Greenhowe

**Affiliations:** 1 Plastic and Reconstructive Surgery, Aberdeen Royal Infirmary, Aberdeen, GBR; 2 Plastic and Reconstructive Surgery, Royal Aberdeen Children’s Hospital, Aberdeen, GBR

**Keywords:** paediatric plastic surgery, rare congenital anomaly, ent surgery, paediatric ent, conchal cartilage, plastic and reconstructive surgery

## Abstract

Anteverted conchal bowl is a rare auricular anomaly in which a convexity of the conchal bowl is seen. The condition may pose both aesthetic and functional implications for affected patients. Most cases are benign and require minimal or no intervention. In severe cases, the external acoustic meatus can be occluded, giving rise to a host of complications. Correction of anteverted conchal bowls is achieved by either conservative or surgical modalities, with the latter commonly undertaken in the post-neonatal stage. Reconstructive surgery can be performed using relatively simple techniques and yields desirable results. Here, we present a case of a patient surgically treated in our Plastic Surgery Unit in the North East of Scotland, along with a description of the operative methods utilised.

## Introduction

In the normally formed external ear, the concha forms a concavity facing anteriorly. Anteverted conchal bowl, also known as an inverted concha, is an uncommon anomaly in which there is an anterior convexity of the concha resulting in a visible aesthetic deformity [1]. Alongside cosmetic implications, severe cases of inverted concha can impact key functions of the ear by occluding the external acoustic meatus (EAM), resulting in complications such as conductive hearing loss, cerumen build-up, and recurrent ear infections due to poor drainage [1,2].

Traditionally, external ear anomalies have been classified into either malformations or deformations [3]. Auricular malformations describe abnormalities arising from an inherent problem of a developing structure and generally develop between the fifth and ninth weeks of gestation [4]. Malformations may result in loss or excess of auricular components [5]. Conversely, auricular deformations are caused when an outside force damages an otherwise normally developing structure and may occur at any point during or after the gestational period [6].

Anteverted concha bowl is considered a deformation abnormality and may present unilaterally or bilaterally [1]. Management options range from conservative measures such as splinting to definitive surgical intervention. Here, we present the case of an eight-year-old female with bilateral anteverted conchal bowls and the surgical technique used for correction.

## Case presentation

Our eight-year-old patient was born at 26 weeks of gestation by emergency caesarean section due to pre-eclampsia and placental abruption. Her neonatal background included patent ductus arteriosus (treated at two months of age), chronic lung disease of prematurity, gastro-oesophageal reflux disease, and 16p13.11 microdeletion, a condition with a variable phenotype and features including developmental delay, cognitive impairment, microencephaly, and seizures.

**Figure 1:**
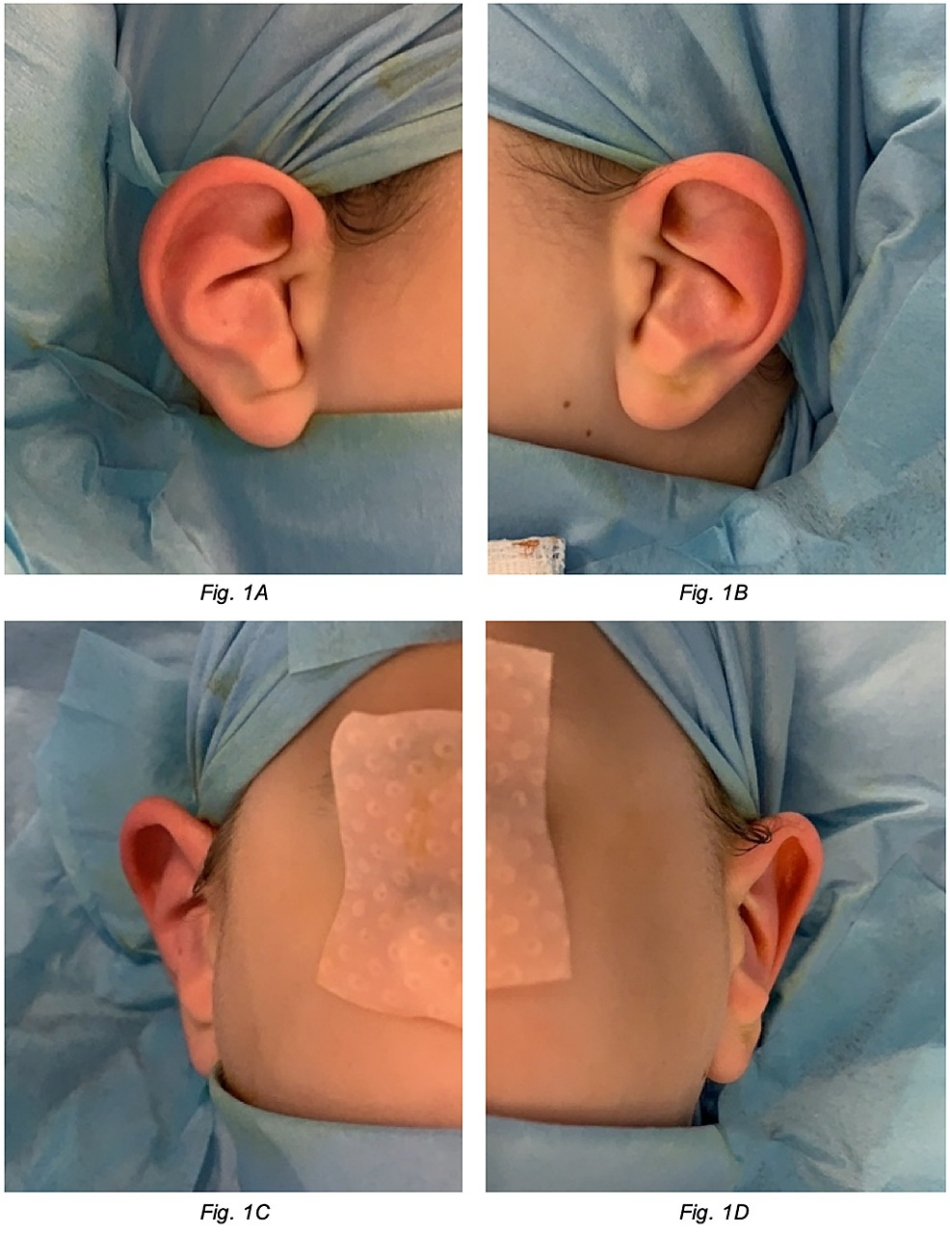
Pre-operative images. (A) Right ear lateral view. (B) Left ear lateral view. (C) Right ear anterior view. (D) Left ear anterior view.

She was under the care of the otolaryngology team on account of her long-established problems with bilateral ear infections and conductive hearing loss. While previous otoscopic examination findings referenced a markedly narrow EAM, it was not until a paediatric otology specialist reviewed her at the age of six that she was identified to have bilateral anteverted conchal bowls. A bone-anchored hearing aid was fitted to the right ear, and she was referred to the plastic surgery team for consideration of correction to improve drainage and reduce the frequency of infections. Figure 1 shows lateral and anterior views of both ears prior to corrective surgery.

**Figure 2:**
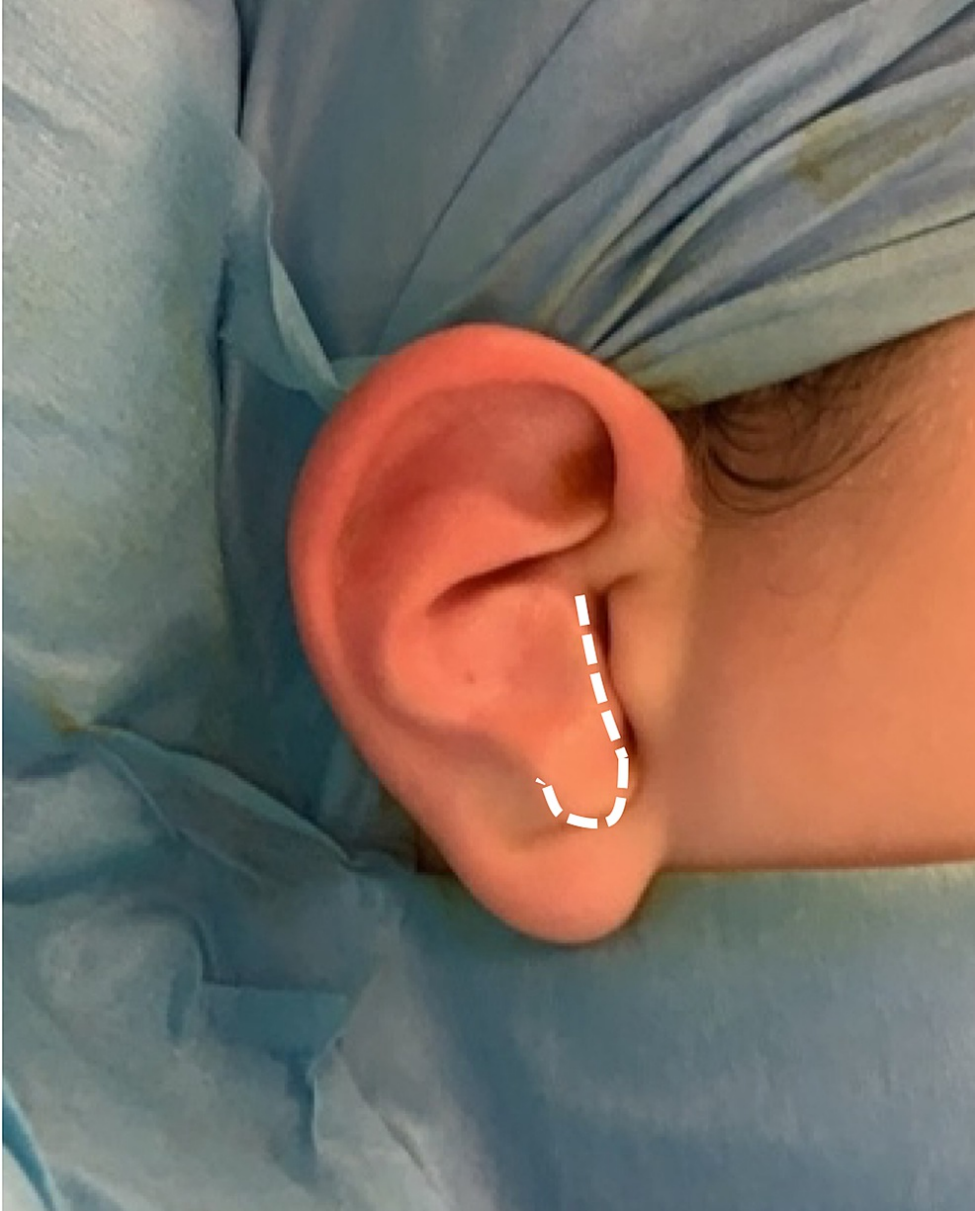
Skin incision.

**Figure 3:**
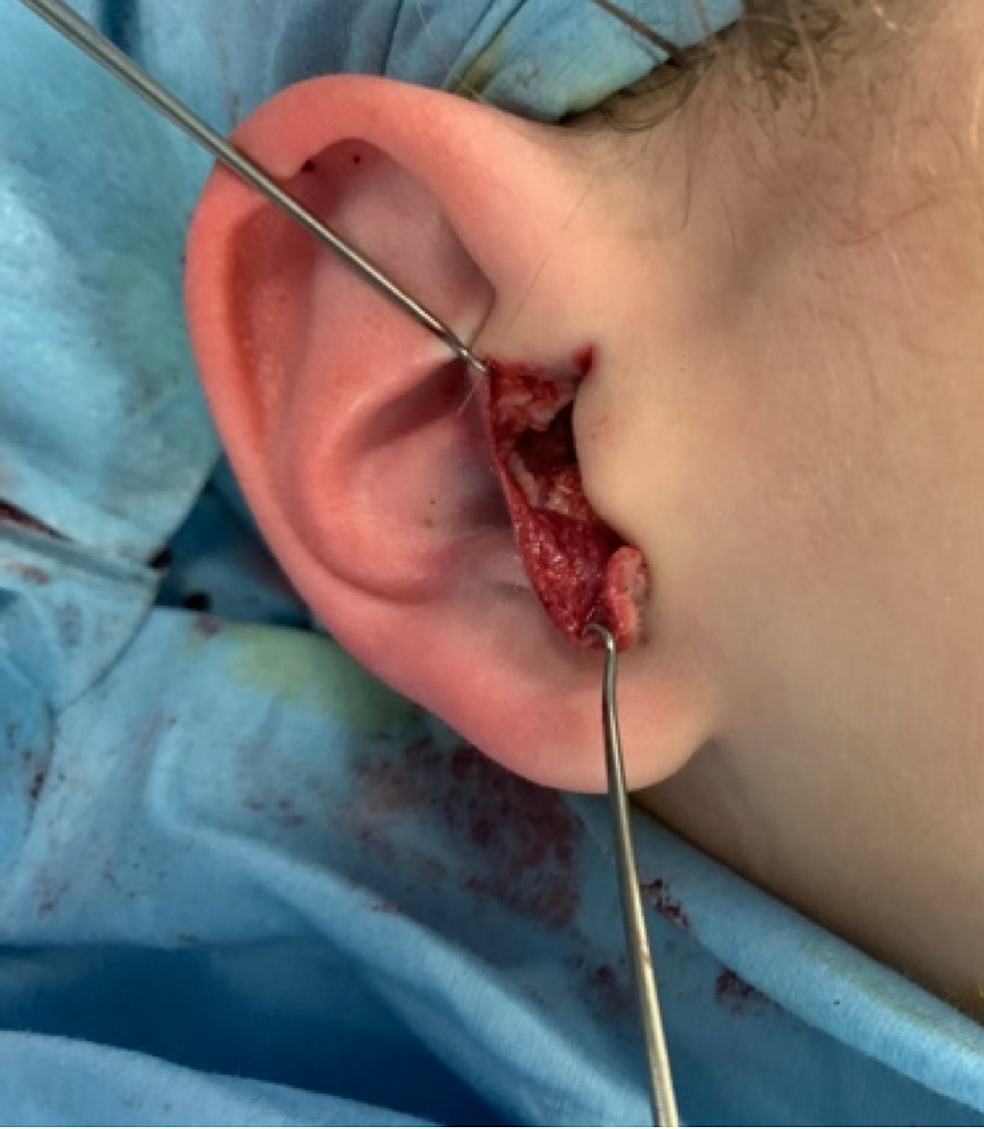
Raising of antitragal skin flaps and flipping of antitragus superiorly.

At eight years of age, our patient underwent correction of bilateral anteverted conchal bowls under general anaesthesia. A skin incision was made over the anterior conchal bowl along the posterior rim of the EAM and extended over the antitragus (Figure 2). Skin flaps were raised over the antitragus to expose the abnormally positioned cartilage which was then incised at the base and flipped superiorly (Figure 3). 4/0 polypropylene sutures were utilised to anchor the antitragus to the conchal base and maintain this position.

**Figure 4:**
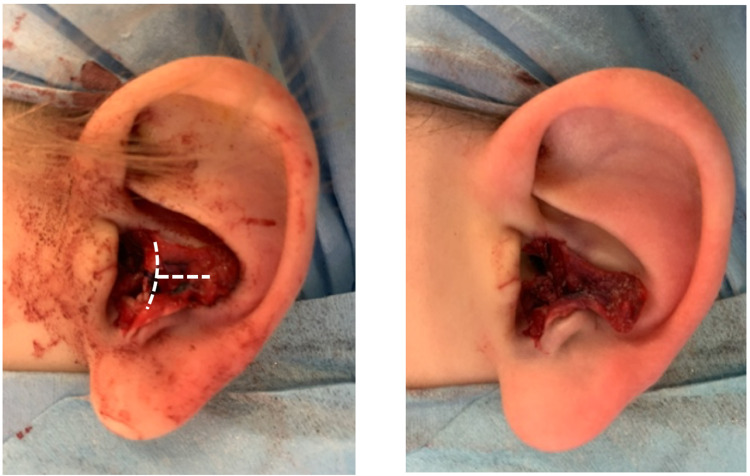
T-shaped incision to flatten and increase the conchal bowl circumference.

Next, a T-shaped incision was made to flatten and enlarge the conchal bowl circumference. The short limb of the T was parallel to the posterior edge of the EAM, and the long limb was then directed away from the EAM into the conchal bowl (Figure 4). This allowed the conchal cartilage to be mobilised as two square flaps which were distracted to increase the circumference. Additional 4/0 polypropylene sutures were placed to anchor the divided cartilages to the deeper tissues.

**Figure 5:**
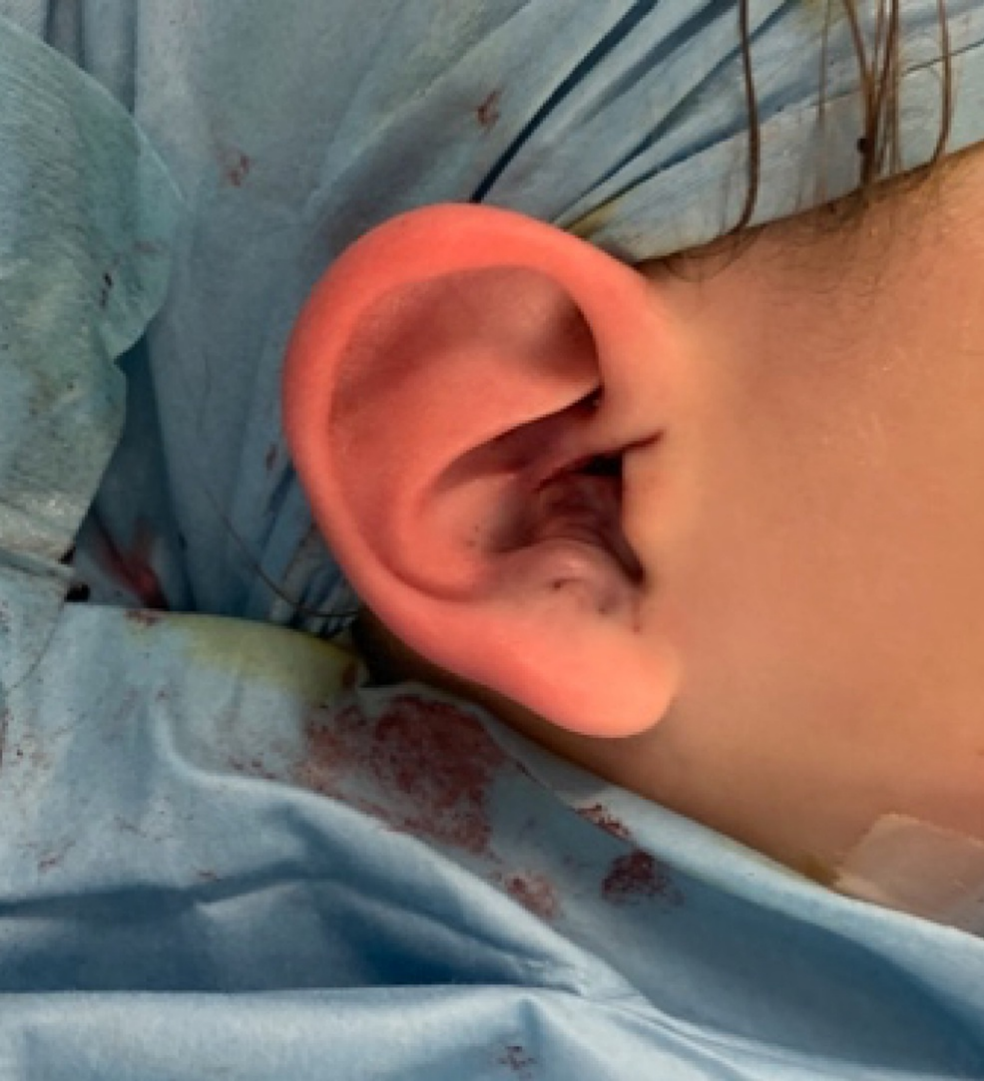
Wounds prior to skin closure.

Figure 5 demonstrates the intra-operative result prior to skin closure, which was carried out using 6/0 polyglactin 910 sutures. The entire procedure was then repeated on the contralateral ear.

The otolaryngology surgeons then examined the ears noting keratinous debris deep within and performed microsuction. Paraffin gauze, mupirocin ointment, and cotton wool were applied directly to the newly retroverted conchal bowls for pressure and a wool and crepe head bandage was used to secure.

**Figure 6:**
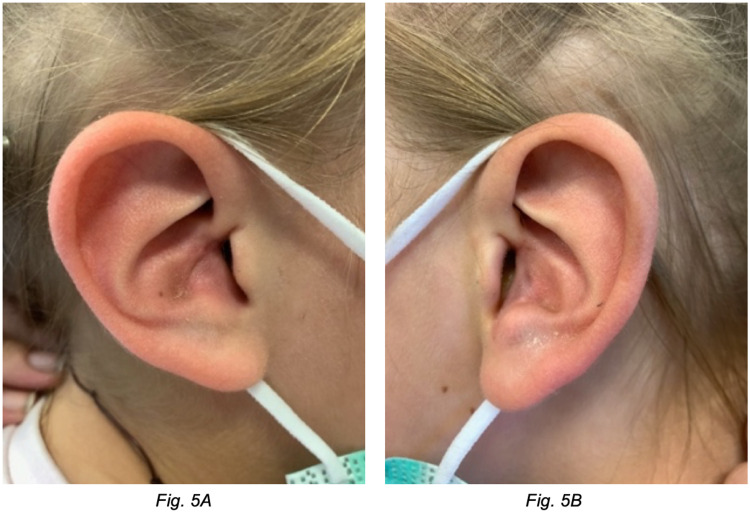
Eight-week post-operative images. (A) Right ear lateral view. (B) Left ear lateral view.

The patient was followed up eight weeks post-operatively. There were no concerns with wound healing and the scars had settled well. There was notably improved opening of the EAM bilaterally, and the aesthetic shape of the conchal bowl cartilage, tragus and antitragus was greatly enhanced with good symmetry. Lateral views of both ears at the eight-week post-operative review are shown in Figure 6.

Following a successful procedure and desirable outcome, she has since been discharged from the Plastic Surgery Department. Because of her intra-operative findings, the otolaryngology team arranged a CT scan which demonstrated findings consistent with bilateral cholesteatoma within the middle ear. She is planned for surgical management of this in the near future.

## Discussion

Auricular deformities are common with 30% of neonates having some degree of auricular anomaly [7]. In a majority of cases, the helix or antihelix is affected [3], and isolated conchal bowl deformity is rare with only a handful of published reports in the literature [1-3,8]. Management among the reported studies varied according to the patient’s age at presentation. A conservative approach was favoured in younger patients versus surgical correction in older patients [1-3,8].

Conservative treatment involves the application of splints or moulds to the external ear. These non-invasive appliances encourage the concha to conform to an adjusted shape. They are made from a non-irritating malleable material and can be custom-made to fit individual patients depending on the deformity. Such appliances perform best in the neonatal period when the cartilage is more pliable [3,5]. Because of decreasing levels of oestrogen, auricular cartilage becomes firmer after birth [4], rendering splinting techniques less successful if delayed [3,5]. However, there is no consensus on a specific age cut-off in the literature. In their study, Tan et al. only recruited patients whose cartilage was malleable enough to allow for digital correction, with an age range of one day to 10 weeks [3], whereas Byrd et al. noted that the efficacy of splinting reduced from over 90% to approximately 50% when commenced after three weeks of life [9]. However, Schönauer et al. reported successful treatment of a three-month-old child with unilateral anteverted conchal bowl with splinting alone but specified that the ear could be manually reshaped with pressure before intervention [1].

For older patients seeking correction of anteverted conchal bowls, for instance, due to functional problems or concerns regarding cosmesis, surgery should be considered. A review of the literature identified seven patients who were managed surgically, with ages ranging from three to thirty-three years [1,2,8,10,11]. Various operative techniques have been described. Some studies reported good results with simple excision of the conchal cartilage [1,10], whereas others would then replace it in a reverse fashion as an autologous graft, thereby reversing the convexity [8,11]. Alternative methods include scoring the inverted cartilage via a posterior approach, reversion, and suturing to the mastoid periosteum to secure [2]. All studies reported good results with these various methods [1,2,8,10,11]. In our patient, a unique method of incising the cartilage was utilised. We found that the T-shaped release of the cartilage increased the circumference of the conchal bowl and allowed for easy manipulation into a corrected position. Auricular support was maintained as there was no cartilage excision, and there was no reliance on revascularisation of a reversed conchal graft as the cartilage flaps remain perfused throughout.

Regrettably, our patient’s condition was not identified until later in childhood, well beyond the window where splinting would be an effective treatment. While our surgical technique was successful in correcting the deformity, had her condition been recognised in early life she could have potentially avoided an operation under general anaesthesia as well as years of recurrent ear infections. Awareness and education of clinicians play a crucial role in early diagnosis and impact available management options as well as outcomes.

## Conclusions

Anteverted conchal bowl deformity is a rare condition that has the potential to impair essential functions of the ear, such as hearing and drainage from within the ear canal. In the neonatal period, splinting is a simple and effective treatment option. In the post-neonatal stage, surgery is the preferred modality of treatment, and desirable results can be achieved with relatively simple techniques. In this report, we have demonstrated successful operative correction using a novel T-shaped release of the conchal cartilage which makes it amenable to manipulation while maintaining auricular support and perfusion to the tissues.

Although considered rare, awareness of this condition is important as the time of diagnosis influences available treatment options. Timely recognition may prevent many years of morbidity.
